# Progress of Veterinary Science

**Published:** 1880-04

**Authors:** 


### PROGRESS OF VETERINARY SCIENCE.

---

26. TRICHINOSIS NOT AS FATAL AN AFFECTION AS IT IS ORDINARILY CONSIDERED.—Drs. Belfield and Attwood, of Chicago, found that eight hogs out of a hundred contained trichinæ, there being about thirty in every cubic inch of muscle. They found that a rat may be fed with a considerable number of trichinæ repeatedly without any disturbance of health, although on killing such subjects of experimental feeding, their bodies were found swarming with trichinæ, one of these animals containing, by approximate calculation, 100,000 of the parasites. They believe that a much larger number of persons are affected with trichinæ than is generally held. Dr. Belfield went so far as to swallow a dozen of living trichinæ, and suffered from no bad symptoms for weeks after. The investigators found that a small quantity of sulphuric acid, so minimal as not to detract from the commercial value of the meat, suffices to kill all the trichinæ contained.—*Boston Med. and Surg. Journal.*

[Editorial Comment.] From the known conditions of parasitic development, the swallowing of a dozen living trichinæ is not to be regarded as an *experimentum crucis.* We have, while engaged in the dissecting room, repeatedly found human bodies full of trichinæ, the subjects having died of different ordinary diseases.

---

27. CONCEALED GLANDERS IN A HORSE—NEGATIVE RESULTS OF AUTO-INOCULATION.—Glanders being detected in one of the horses belonging to a brick-yard proprietor near Munich, this animal was killed and the others isolated. No untoward symptoms being observed, the interdict was raised after the customary period. *Two years after,* no other horses being added, another horse died from an acute affection, and the post-mortem revealed the presence of *chronic glanders.* The discovery of this fact led to a renewed activity on the part of the veterinary police. The fellow of this horse was found to have a slight glandular swelling on the left side of the larynx ; there was a secretion of clear mucus, chiefly from one nostril ; percussion of the sinuses showed nothing abnormal ; the animal was entirely free from fever, had an excellent appetite, and was in good spirits ; the skin did not show the normal gloss. On inoculating the animal with the nasal secretion, the result was entirely negative. After a long and thorough examination, and a prolonged period of observation, Prof. Friedberger found a cicatrical spot high up on the nasal septum, with the rhinoscope. Trephining the maxillary sinus revealed nothing abnormal. Attempts to inoculate this wound, both with the transparent and *starchy* as well as with a clump of thick muco-purulent matter which was thrown off from the right nostril, failed. The animal was killed, glanders being suspected on the strength of the above-mentioned cicatrix. Prof. Bollinger made the post-mortem and found chronic glanders of the nasal cavity, large stellate cicatrices, perforation of the nasal septum, miliary nodules (see Archives, Jan., 1880, p. 36), in the perilaryngeal glands, glanders of the larynx, trachea and bronchi, bronchiectasis, pulmonary glanders of both sides, cicatricial athrophy of the left lung.

There can be no doubt that this animal had been glandered two years before, that no symptoms indicated the disease, and that the apparently healthy animal constituted a possible source of renewed infection. The case also showed that auto-inoculation cannot settle the question of the existence of glanders.

On inoculating several rabbits, glanders was produced in two, death took place by ordinary sceptic infection in a third, while the result in a fourth was entirely negative. —*Yahresbericht d. K. Th. Arznei. Schule in Munchen*, 1877–1878.

---

28. Inoculation of Cattle for Pleuro-pneumonia.—Mr. Reid, V. S, Inspector for Leith, read a paper on this subject, in which he expresses opinions quite at variance not only with some of his colleagues but also with the most prominent authorities of Germany and the United States. However, his views are advanced upon a basis of statistics which cannot be entirely overcome, and although we would strongly endorse the stamping-out process for such countries as America, as long as the disease can be confined to narrow quarters, yet, if the conclusions of Mr. Reid are correct, the process of inoculation deserves consideration as a means for checking the ravages of the disease where "stamping out" would prove ruinous and impracticable. He censures the Act of 1878, which provides that diseased cattle shouid be slaughtered and others isolated for fifty-six days, justly, because the infection may reside in latent cases for three months, and even much longer. Mr. Rutherford reintroduced the system of inoculation at Leith, and the following statistics there are exhibited by Mr. Reid, as speaking for themselves. In 1874, out of 530 cows, 155 were effected with pleuro-pneumonia; in 1875, 90; in 1876, 107; in 1877, 90—showing an average of 110 animals brought to the slaughter-house with the disease. After introducing inoculation in 1878, only 38 were effected, and even this mortality Mr. Reid attributes to the negligence of those who accepted inoculation, only when the disease had already manifested itself, as shown by the fact that of the 38 cases, 26 were confined to 13 "byres," occupied by 138 cows. In the discussion Professor Walley made the rather damaging statement that in Holland, where inoculation was compulsory, the disease still prevailed. In reply, the chairman, Mr. Rutherford, offered to arrest any outbreak in London at his own expense.—*The Veterinarian, Nov.*, 1879.

---

29. Absence of One Kidney in a Mare.—Mr. Stanley, F. R. C. V.S, relates a case of enteric disease in a mare, which running an unfavorable course, led him to recommend the destruction of the animal. On the autopsy it was discovered that while the right kidney was considerably hypertrophied (also containing four small calculi) the left was entirely absent.—*The Veterinarian, November*, 1879.

---

30. On Intestinal Torsion and Displacement of the Colon in the Horse. Before the Scottish Metropolitan Veterinary Association, Professsor Walley read a paper, of which we present a full abstract in justice to its practical and important character. He treats—1st. Of torsion, or displacement of the double colon with venous (partial vascular) strangulation. 2d. Of torsion of the double colon with arterio-venous (complete vascular) strangulation. 3d. Of displacement of the double colon sigmoid flexure—in an oblique forward direction from left to right, and across the small intestines. He claimed to be able to diagnosticate these affections with " almost unerring certainty " All these affections are more frequent in heavy than in light horses, because the bowels are more voluminous in the former, and more largely filled with food and water. The actual displacement takes places more frequently in consequence of the following causes: 1. By rolling about in the agonies of colic. 2. By the powerful contraction of the muscular walls in spasms of the gut. 3. By the action of gas on the interior of the gut when it is comparatively empty; the power of imprisoned gas to displace a knuckle of intestine is well and often seen in making autopsies of horses whose intesties have become inflated with gas from decomposition of their contents after death. The sympsoms of torsion and displacement are usually not observed for several hours. Of the three forms mentioned, the second produces its symptoms most rapidly. Often they are preceded for some time by enteritis or prolonged enteralgia. In the first form, the previously full and irritable pulse becomes softer, the injected mucous membranes paler, the body moist, countenance anxious, breathing hurried and labored, head turned wistfully to flank; the enteric pains are subdued, though still persisting; if attempts are made to distend the jugular vein for purposes of bleeding, is will be found impossible to render it tense, difficult to drive the flam into it, and when opened only a small quanity of blood, and that usually of a dark color, can be abstracted. Temperature usually below 104° F., and on exploration a resilient tumor can be felt *per rectum*, so may also be felt the tense and twisted longitudinal bands of the gut. Death in 12–16 hours. The part of the bowel below the torsion is usually emptied rapidly, the fæces being pultaceous and passed in small quantities. After death, sero-sanguineous effusion is usually found in the peritoneal cavity. The occluded intestine enormously increased in bulk, weight and thickness. Peritoneum sometimes ecchymosed. On cutting into the occluded gut, fœtid gas escapes. The line of demarcation between the deceased and the healthy part of the gut is well pronounced.

In the second form, the symptoms are developed with suddenness, except, of course, where enteritis has preceded; the pulse is bounding and almost incompressible; the veins are full of quite bright blood; venesection only slightly reduces pulse, mucous membranes injected, perspiration profuse, tremors marked, eye prominent, countenance desperate, pain persisting and agonizing. Death in from 4–6 hours. On autopsy, the gut is found mortified, the veins and arteries plugged.

In the third form the symptoms are slowly developed; it is not a common affection, the writer having had his attention called to but two cases. The pain is subdued, pulse quick and weak, membranes injected, breathing labored, temp. 103 F., œsophageal regurgitation of fluid and gas, great distension of œsophagus with each regurgitation, leading to the supposition that the animal is suffering from impaction of that tube. There will be either vomiting or attempts to vomit.

Post-mortem: the colon may be healthy, small intestines present punctiform hemorrhages, gastric mucous membrane inflamed; this latter is secondary, however; alimentary canal distended with gas.

The writer does not agree with Mr. Bouley that death takes place in either form from putrid infection, and advances some cogent reasons for so differing.

He then adds some remarks on the use of the trocar and canula in tympany. At times great benefit and immediate relief are obtained; at others, although over a dozen punctures are made, the operation fails. Prof. Walley concludes that relief will be most certain where the tympany is circumscribed and due to decomposition of food. He also finds that many failures are due to the shortness of the canula. He now uses a curved trocar and canula, a quarter of an inch in diameter and seven inches long; the advantage of the curve is that ingesta do not so rapidly block the passages, and that it is not so apt to be displaced by the intestinal peristalsis as the straight instrument.

"Introducing the trocar and canula when the animal is on its back or side, it must be borne in mind that the relative position of the ingesta and the gas are altered, the former gravitating to the lower part of the intestinal cavity, the latter ascending to the upper; due allowance must also be made for the thick layer of abdominal fat which is always present in fat animals. The removal of the hair and the making of a small incision in the skin, facilitate the introduction of the trocar; but in making this incision an unlooked-for accident may happen, viz., the infliction of a large wound by the sudden contraction of the muscles at the moment the lancet was introduced; hence the necessity of gathering up a small fold of skin, where practicable, between the finger and thumb, before making the incision. . . . . . . . . . . . If necessary, the tapping should be repeated again and again, and in different situations. I have never yet seen any ill results follow the punctures, and if any hesitation is felt the small trocar and canula attached to the hypodermic syringe may be first introduced."—*The Veterinarian, November*, 1879.

---

31. WOUNDS OF THE ARTICULATIONS OF THE HORSE—THEIR TREATMENT.—It is observed that the clean wound which results after the cauterization of any wound which communicates with the joint, has a great tendency to undergo rapid cicatrization. It is the fragments of disorganized or half living tissues which complicate the healing process, cauterization of these parts by facilitating their separation, therefore hastens the healing process. The acid nitrate of mercury is an excellent means for this end, but should be employed with prudence and by an expert hand.—*Des Plaies Articulaires. C. Dubois Ann. de Médecine Vétérinaire, Fevrier*, 1880.

---

32. HYDROPHOBIA TRANSMITTED FROM MAN TO THE RABBIT.—From St. Louis *Clinical Record*, December, 1879: M. Maurice Raynaud communicated to the Academie de Médecine a case of hydrophobia in a human subject, from whom, on the eve of death, saliva and blood were taken to inoculate rabbits with. The inoculations with blood failed to produce any result (!); those with saliva produced hydrophobia within a few days. The submaxillary glands of two "rabid" rabbits were equally potent in transmitting the disease to other rabbits. It is to be inferred, therefore, M. Raynaud concludes, that rabies might be transferred from man to man by the bite.—*Le Progrés Médicale, November, 1879.*

---

33. THE RELATION BETWEEN GLANDERS AND PYAEMIA.—Professor Maury calls attention to some facts, which, though one might almost consider them to be truisms, yet do not appear clearly appreciated by some veterinarians. His conclusions are given in the *Belgian Annals of Veterinary Medicine*, from the "Veterinary Review," of Toulouse, as follows: 1. Leucocytosis is not peculiar to glanders. 2. It is symptomatic of many other complaints, such as purulent infections. 3. Even in glanders it occurs only in the very advanced stages of the malady. 4. It should not be considered as a means of diagnosis for latent glanders.—*Revue Vétérinaire de Toulouse, Nr., 1878.*

---

34. CONSTRICTION OF ANUS IN A BULL.—Mr. Molinié describes a case where a young bull was much distressed at every evacuation by a congenital constriction of the anus. Subcutaneous division of the sphincter ani gave complete relief.—*Revue Vetérinaire de Toulouse, Nr. 12, 1878.*

(Reported by Prof. Dessart, in the Belgian Annals of Veterinary Medicine.)

---

35. CHICKEN CHOLERA.—In the so-called "cholera" of fowl, the characteristic symptoms are as follows: The wings droop, an unconquerable somnolence is noted; on startling the animals and forcing them to open their eyes, they seem to wake as from a deep sleep, and soon the lids close again, and usually death occurs without any particular movement of the animal, and after a mute agony; at most its wings flutter a little as it dies. The disease is produced by a microscopic organism which has been successively described, more or less perfectly, by Moritz of Alsatia, Peroncito of Turin, and Toussaint of Toulouse. This microscopic organism differs from allied ones, by not developing in beer; in fact, this fluid, which favors the development of the anthrax bacteria, is fatal to it. Animals recovering from one attack, present an immunity to it in future; the Cochin-China breed presents a natural immunity to the virus.—*Pasteur, report in L' Union Médicale, November 18, 1880.*

---

36. TREATMENT OF BLOOD FILARIA.—The most effective remedy in the treatment of blood filaria in man, is turpentine, not only on account of its diuretic effect, but also as a parasiticide.—*Dr. Abbe, N. Y. Med. Journal, February 1880.*

---

37. THE PATHOLOGICAL HISTOLOGY OF EPIZOOTIC PLEURO-PNEUMONIA.—There are three stages, according to Dr. Roy, in the lung changes of this disease. I. Stage of infiltration by a semifluid, gelatinous exudation. II. Stage where this exudation is more or less solidified. III. Where the diseased tissue is paler, firmer and dryer, the blood-vessels having become impervious. These stages are arbitrary, for lung tissue in one stage may merge gradually into lung tissue of an earlier or later stage of disease. The exudation is most usually in the interlobular connective tissue. The morbid tissue from its relation to the normal and improvised lymph-channels, renders the term *lymph-adenoid* descriptive of the structure. The subpleural exudation differs from the interlobular in the greater tendency to cicatricial change. The laminated connective tissue, formed from the subpleural exudation, is of that kind which has been termed *keratoid connective tissue* by Köster; it should not be confounded with the frequently overlying pleuritic false membrane. Unless the blood-vessels are the seat of infarctions, their coats show no noteworthy change.—*Charles S. Roy, M. D. The Veterinary Journal, January, 1880.*

---

38. Further Facts relating to Toxaemic Haemoglobinuria of the Horse.—As a frequent complication, but bearing no constant relation to this disease, Prof. Bollinger found embolic occlusion of the renal vessels, causing hemorrhagic nephritis, the kidneys being changed into a bloody broken-down mass. The muscles in one case were very fragile, and this condition is found well marked in the masseter, tetanus having, however, occured as a complication.—*Yahr. Ber. d. K. Central. Th. Arz. Schule, 1879.*

---

39. Microscopic Organisms in their Relations to Disease.—Prof. Greenfield, adopting a theory somewhat at variance with the views expressed in one of our editorials, in a very interesting series of lectures delivered at the Brownian Institute, claims that Micrococci and Bacteria are distinct forms, and that specia forms are characteristic of special diseases; thus *Micrococcus vacinæ* has been supposed to be pathogenic of cow-pox, and *M. diphtheriticus* of diphtheria [in neither case properly established.—Editor].

A filamentous bacterium, the *Bacillum anthracis* is considered pathogenic of anthrax, and a similar form is claimed to exist in farcy. The difficulty under which those who defend the germ theory labor is well illustrated by Prof. Greenfield's statement, that "error is liable to arise from confusion of harmless bacteria with the virulent forms."

